# HPV Vaccination: Polish-Language Facebook Discourse Analysis

**DOI:** 10.3390/ijerph19020914

**Published:** 2022-01-14

**Authors:** Karolina Sobeczek, Mariusz Gujski, Filip Raciborski

**Affiliations:** 1 Faculty of Health Sciences, Medical University of Warsaw, 02-091 Warsaw, Poland; 2 Department of Public Health, Medical University of Warsaw, 02-097 Warsaw, Poland; mariusz.gujski@wum.edu.pl; 3 Department of Prevention of Environmental Hazards and Allergology, Medical University of Warsaw, 02-091 Warsaw, Poland; filip.raciborski@wum.edu.pl

**Keywords:** HPV vaccination, social media, Facebook, COVID-19

## Abstract

Social media platforms are widely used for spreading vaccine-related information. The objectives of this paper are to characterize Polish-language human papillomavirus (HPV) vaccination discourse on Facebook and to trace the possible influence of the COVID-19 pandemic on changes in the HPV vaccination debate. A quantitative and qualitative analysis was carried out based on data collected with a tool for internet monitoring and social media analysis. We found that the discourse about HPV vaccination bearing negative sentiment is centralized. There are leaders whose posts generate the bulk of anti-vaccine traffic and who possess relatively greater capability to influence recipients’ opinions. At the beginning of the COVID-19 pandemic vaccination debate intensified, but there is no unequivocal evidence to suggest that interest in the HPV vaccination topic changed.

## 1. Introduction

Human papillomavirus (HPV) causes infections of the skin and mucous membranes [1]. Most HPV infections are sexually transmitted, asymptomatic and resolve spontaneously. Highly oncogenic strains of HPV are responsible for the development of precancerous and cancerous lesions of the uterine cervix, anus, penis, vulva, vagina, oronasal space and other sites. Over 200 types of HPV are distinguished, of which 13–15 are regarded as oncogenic [2,3,4]. Types 16 and 18 are responsible for approximately 70% of cases of cancer of the uterine cervix in the world [5]. An ICO/IARC HPV Information Centre report states that cervical cancer (CC) is the third most frequent cancer among women [6]. According to data from the Polish National Cancer Registry, about 2500 women in Poland develop cervical cancer annually, with about 1600 annual deaths. In 2018, the standardized cervical cancer rate (ESP2013) per 100,000 population was 11.4 for incidence and 7.7 for mortality [7].

According to the World Health Organisation (WHO), HPV vaccines (primary prevention) combined with secondary prevention will allow for eliminating most cases of CC [8]. Although HPV vaccination is especially beneficial given before potential exposure to HPV through sexual contact, studies show that it is also effective in sexually active women and, in the adjuvant setting, reduces a risk of recurrent cervical dysplasia after surgical treatment [9,10,11,12,13]. Vaccines against HPV of proven efficacy and safety have been approved for use in European Union countries since 2006 r [14,15,16,17]. Due to vaccination campaigns in high-income countries over 5–8 years, the prevalence of HPV 16 and 18 fell by 83% in the population of girls aged 13–19 and by 66% among women aged 20–24 years [18]. In Poland, HPV vaccination with 2-, 4- and 9-valent vaccines is recommended, non-mandatory, offered at a price in primary health care and free of charge under local government health-promotion programs. A total of 2.05% of 10–14-year-old girls were vaccinated through these programs in the years 2009–2016 [19]. In Poland HPV vaccination is not included in a national immunization program. Therefore, complete HPV vaccination coverage statistics are not available. A report from the Polish Agency for Health Technology Assessment and Tariff System (AOTMiT) based on data from the National Institute of Public Health-State Institute of Hygiene (NIZP-PZH) and the Central Statistical Office (GUS) shows that HPV vaccine coverage in the female population under 19 years of age in Poland was approx. 1–1.5% in the years 2015–2017. However, AOTMiT, without quoting any data sources, estimates overall coverage to be higher [20].

The Internet is a potential source of knowledge about HPV vaccination [21]. A study by the Centre for Public Opinion Research (CBOS) showed that 68% of adult Poles went online at least once a week in 2020, and nearly half of them had social website accounts [22]. A July 2020 report by the research company Gemius states that Facebook had 20,888,772 users in Poland, or 54% of the total population (including children) and shows that in Poland Facebook is the most popular social websites, more popular than Twitter [23]. Analyses of vaccine-related content available on social media platforms have been conducted in the world. Those analyses are based on data extracted from social media platforms and examine vaccine hesitancy, acceptance, sentiment and administrative burden [24,25,26,27,28]. Study data acquisition from Facebook and Twitter commonly relies on automated IT solutions offering data collection, monitoring and analysis [29,30]. Topics regarding vaccination include HPV vaccines [31,32,33]. To examine the state of vaccine confidence in Poland, the cross-sectional study was carried out using the computer-assisted web interview (CAWI) technique, and showed relatively high confidence in mandatory vaccination among adults [34]. Almost 75% of the respondents agreed or strongly agreed that mandatory vaccinations are safe. The study reported that participants aged 25–34 years were the least trustful of vaccinations. Studies that examined HPV vaccine acceptance and hesitancy in Poland demonstrated fear of side effects, lack of trust in vaccination effectiveness and insufficient knowledge as the main reasons for vaccination refusal, and vaccination availability, the price of the vaccine, the need to repeat the vaccination (compliance) and concern about sexual promiscuity as other barriers [35,36,37].

Following the emergence of SARS-CoV-2 virus in December 2019 [38], a growing number of cases of coronavirus disease and the proclamation of a pandemic, there are ongoing efforts in the world to effectively counteract this global threat [39]. In Europe, the first laboratory-confirmed case of COVID-19 was registered on 24 January 2020 [40]. In Poland, it occurred 40 days later (4 March 2020) [38]. According to statistics published by the Polish government by 26 November 2021, 3,461,061 cases of coronavirus infections and 82,607 related deaths have been registered [41]. A vaccine against the new coronavirus appears to be a potential solution for controlling the pandemic [42]. Poland is one of the countries with the lowest vaccination rates against COVID-19 in European Union. By the end of November 2021 less than 55% of the total population has taken at least one dose of the COVID-19 vaccine [43]. The global debate on vaccination, including HPV vaccination was influenced by the pandemic [44]. However, there have been no publications investigating the relationship between the intensity of the anti-HPV debate and the COVID-19 pandemic in Poland.

The main objective of this paper is to characterize Polish-language HPV vaccination discourse on Facebook over 30 months (between 1 January 2018 and 30 June 2020). Intermediary objectives are: (1) to characterize the topic range, (2) to assess HPV vaccination sentiment and (3) to trace the possible influence of the COVID-19 pandemic on changes in the HPV vaccination debate. Identification of the factors associated with different attitudes and beliefs toward vaccines may help establish best practices in the promotion of vaccination.

## 2. Materials and Methods

A quantitative and qualitative analysis of HPV-related online content in Polish was carried out based on data collected with the SentiOne (SentiOne, Gdańsk, Poland) [45] tool for internet monitoring and social media analysis. Study data were acquired as a result of four queries exploring the following topics: (a) HPV vaccination, (b) vaccination in general, (c) HPV vaccination but without posts concerned with the coronavirus pandemic and (d) vaccination in general without posts concerned with the coronavirus pandemic. The keywords for the individual queries are listed in Table 1. A keyword ending in an asterisk (“*”) embraces that word with appropriate grammatical endings added.

The posts included in the queries were public, written in Polish and posted on Facebook between 1 January 2018 and 30 June 2020. The study data were exported to a Microsoft Excel spreadsheet. Individual entries comprised the content of the post, link to the post, date of posting, number of reactions (like, love, wow, haha, sad, angry and thankful), comments and shares, and the author’s ID number.

**Figure 1:**
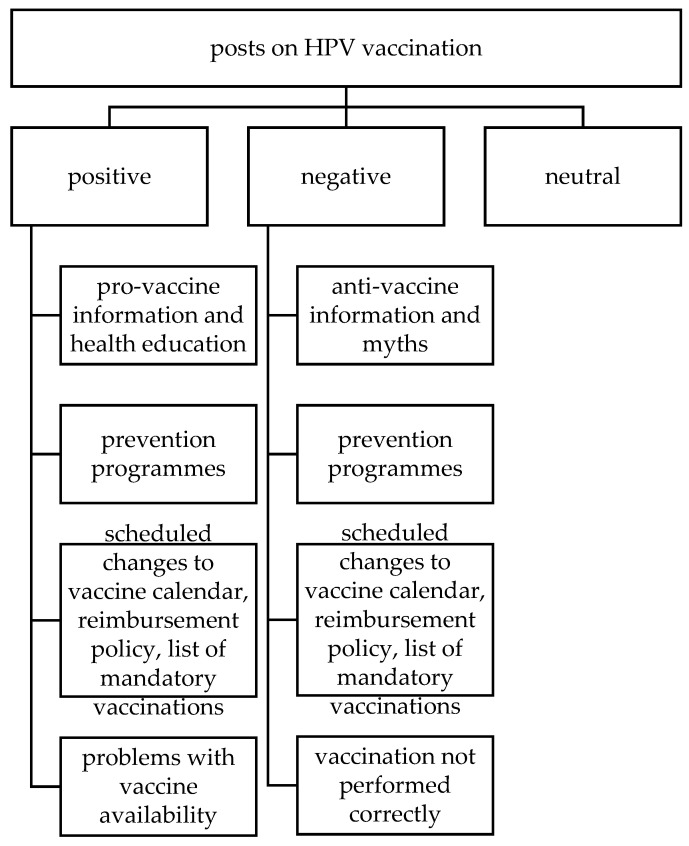
Post categories for detailed analysis.Source: original materials.

A qualitative analysis was conducted with MAXQDA 2020 software (VERBI Software, Berlin, Germany) [46] on a sample of 100 posts about HPV vaccination with the highest engagement (E) scores. Engagement was defined as the sum total of the reactions, comments and shares generated by a post [30,47]. The content of posts was analyzed together with the content of the links included in a post. Posts were categorized manually by a researcher with regard to the expressed sentiment towards HPV vaccination (positive/negative/neutral) and the discussed topic [25]. To improve the accuracy, every post was categorized twice and then in case of a disagreement the third, final categorization was conducted. Sentiment was defined as positive when the post and/or linked content contained information about benefits from vaccination (e.g., “the vaccine protects”) or a positive opinion (e.g., “it is worthwhile to get vaccinated”, “I get vaccinated”) or information about the availability of prevention programs. Negative sentiment was identified when the post and/or linked content contained content discouraging vaccination (e.g., “she died after getting her vaccine”, “pharmaceutical companies lie”, “think twice whether you want to place your child in danger”). Neutral sentiment was identified if the information provided was ambiguous and/or not evaluable. The topic-related categories were determined based on the information that post and/or linked content regard and are presented in Figure 1.

We assumed that pro-vaccination content is such that expresses positive sentiment towards vaccination and is consistent with contemporary medical knowledge. Posts expressing the opposite view were defined as anti-vaccination. Analysis of posts expressing negative sentiment took into account their authors. For the purposes of the study, the author of a post was defined as the page that the post originated from. The type of content in a post was also classified into the following categories: text, external link, photograph and video clip.

A quantitative analysis compared changes in monthly posting counts across the four queries. As the period analyzed included the initial phase of the COVID-19 pandemic, attention was paid to mentions of the pandemic and the effect of the pandemic on interest in vaccination in Poland.

## 3. Results

**Figure 2:**
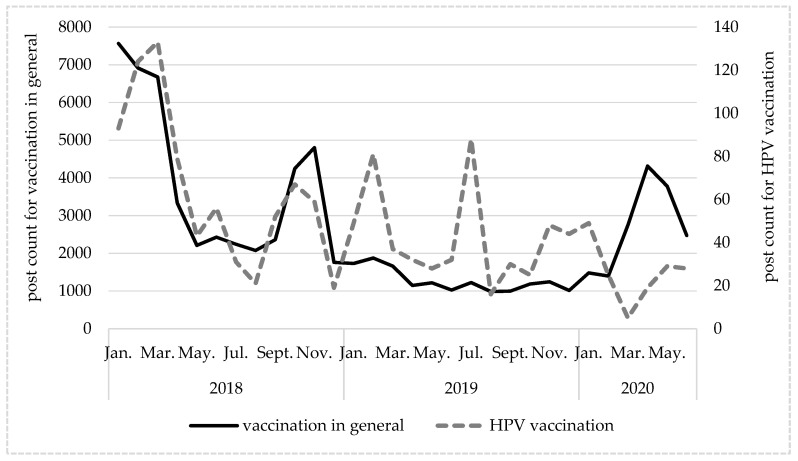
Changes in numbers of posts in Polish about vaccination in general and anti-HPV vaccination over time. Source: original materials.

**Figure 3:**
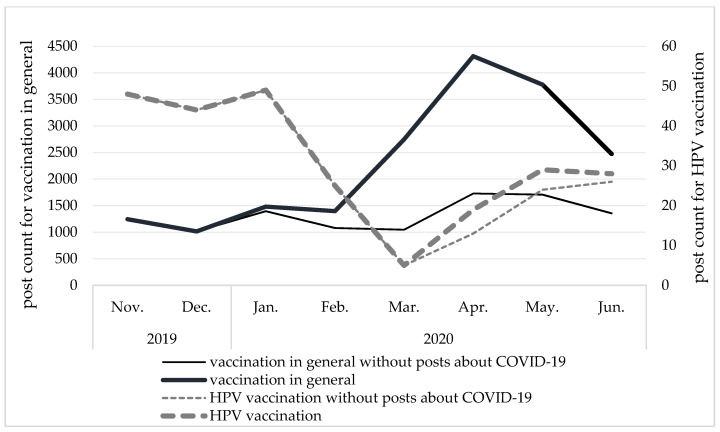
Influence of COVID-19 pandemic-related topics on number of posts in Polish about vaccination in general and anti-HPV vaccination over time. Source: original materials.

In 2018, publication trends followed a similar pattern for posts on vaccinations in general and posts on HPV vaccination, but there were substantial numerical differences. Posts about HPV vaccination peak in March, at 133. For comparison, there were 6675 posts regarding vaccination in general in that month. In 2019, there were several peaks in the count of posts regarding HPV vaccination, and they were not paralleled by increases in the number of posts regarding vaccination in general. In 2020, when topics related to COVID-19 coronavirus were gaining in popularity, the number of posts regarding vaccination in general increased and the number of posts regarding HPV decreased. In April 2020, the number of posts about vaccination reached its maximum, with 60% of the posts referring to coronavirus. These findings are summarized in Figure 2 and Figure 3.

In the sample of 100 posts selected for detailed analysis, the sentiments expressed in 50 of them were classified as negative, with 47 classified as expressing a positive sentiment and 3 expressing a neutral sentiment. The posts contained text (*n* = 96), external links (*n* = 73), photographs (*n* = 23) and video clips (*n* = 6). Negative posts were submitted by 7 authors. The number of posts that each author submitted equals: *n*_1_ = 22, *n*_2_ = 16, *n*_3_ = 4, *n*_4_ = 4, *n*_5_ = 2, *n*_6_ = 1 and *n*_7_ = 1. Nearly all of the negative posts contained anti-vaccine information/theories (*n* = 45). Some referred to scheduled changes to the vaccine calendar, reimbursement policy and list of mandatory vaccinations (*n* = 10), prevention programs (*n* = 4) and situations where vaccination was not carried out correctly (*n* = 4). Most posts expressing positive sentiment to vaccination spread pro-vaccine and/or health education messages (*n* = 42), other topics including prevention programs (*n* = 32), scheduled changes to the vaccine calendar, reimbursement policy and list of mandatory vaccinations (*n* = 14) and problems with commercial availability of vaccines (*n* = 9).

The post with the highest engagement score (E = 10,727) is a positive post that generated 8927 reactions, 887 comments and 913 shares. The post was submitted by a presidential candidate and included an announcement that there are plans to introduce a program of universal HPV vaccination for women and young boys. The following four posts with the highest E scores (4668, 4584, 4567 and 3442) expressed negative sentiment. The lowest E score in the sample of 100 posts was 547. The median E score for negative posts was 862, compared to 908 for positive posts. Mean E scores were 1284 for a negative post, 1326 for a positive post and 721 for a neutral post. The respective mean numbers of comments were 189, 274 and 149. Positive posts generated an average of more than twice as many reactions than negative ones. In both cases, “like” was the most popular reaction. Positive posts generated higher percentages of “love” reactions and lower percentages of “angry” reactions compared to negative posts. These findings are presented in Table 2.

## 4. Discussion

### Key Results

Facebook users are more interested in posts expressing positive sentiment to vaccination than negative ones. The most popular topics of posts regarding HPV vaccination, besides disseminating pro- (42%) or anti-vaccine (45%) information, comprise prevention programs (36%) and scheduled changes to the vaccine calendar, reimbursement policy and list of mandatory vaccinations (24%). Two authors are responsible for the publication of 76% of the most popular public Polish-language posts expressing negative attitudes towards vaccination. At the beginning of the COVID-19 pandemic vaccination debate intensified, but there is no unequivocal evidence to suggest that interest in the HPV vaccination topic changed.

Paguio et al. investigated the effect of the COVID-19 pandemic on interest in vaccination in the world and found a positive correlation between the pandemic and interest in influenza and pneumococcal vaccines and a negative one regarding HPV vaccination [42]. The peak of interest in the former two vaccines was in February and March. In Poland, the onset of the coronavirus epidemic was delayed a few weeks compared to most other European countries, which may be the reason behind a peak of interest in vaccination showing itself in April in our study. The CBOS report states a definite increase in interest in influenza vaccination in the autumn and winter of 2020 compared to previous years. The report also states that, should a COVID-19 vaccine become available, 36% of the respondents would get vaccinated, and 47% did not intend to do so [48]. The popularity of topics related to prevention programs and scheduled changes to the vaccine calendar, reimbursement policy and list of mandatory vaccinations noted in our qualitative analysis may be related to Poland being the only EU country besides Romania where HPV vaccination is not state-funded [49]. Announcements of free-of-charge vaccination campaigns are posted to social media websites to promote pro-health programs arranged by local government units.

Phrases indicating unambiguously what kind of sentiment towards vaccination a post is expressing were present in 97% of the posts. There were 3% more negative posts than positive ones. In an analysis of French-language Twitter posts, Gargiulo et al. noted greater activity of posters expressing anti-vaccination views than those with a positive attitude [24]. This was also observed in a study of the degree of polarization of the sentiment towards vaccination among Facebook users posting in English [50]. The small difference between the number of negative vs. positive posts in our study may be secondary to the procedure for selecting posts for analysis, based on an engagement score. Hoffman et al. noted that posts promoting HPV vaccination stir a debate with negative views expressed [51].

The main limitation of the method employed in our study is that our analysis was based on the sentiment expressed by active Facebook users who make their posts public. It is possible that the distribution of positive vs. negative sentiment is different in private comments and groups. The same objection can be raised with regard to individuals without online access and/or access to Facebook. Even though the analytical tool we used offers a functionality that automatically classifies mentions, in order to reduce the risk of misinterpretation, we decided to assign posts to attitude categories manually. For the qualitative analysis, the sample was not random, but was based on so-called engagement, because we were interested in measuring the reach and influence of posts’ content on recipients. Hence, analysis concerned posts that highly engaged the readers.

## 5. Conclusions

1. The discourse about HPV vaccination bearing negative sentiment is centralized. There are leaders whose posts generate the bulk of anti-vaccine traffic and who possess relatively greater capability to influence recipients’ opinions. The identification of these individuals and organizations and their “modes of action” creates a possibility of limiting the reach of their accounts by social platforms moderators in order to prevent fake-news dissemination. Moreover, understanding the type of content used for promoting anti-vaccine information may later help in establishing a narration and implementing future health education programs.
2. The discourse bearing positive sentiment towards vaccination is decentralized. Promoting leaders who disseminate information consistent with state-of-the-art medical knowledge and foster knowledge about vaccination is worth considering in terms of health policies.

## Figures and Tables

**Table 1:** SentiOne-supported queries. Source: original materials.

No.	Posts About	Search Keywords	Excluded Keywords	Number of Posts
1.	HPV vaccination	szczepi * [vaccine *] hpv, szczepi * wirus * brodawczaka ludzkiego [human papillomavirus], cervarix *, gardasil *, silgard *	-	1442
2.	Vaccination in general	szczepi *	-	78,124
3.	HPV vaccination without posts related to the COVID-19 pandemic	szczepi * hpv, szczepi * wirus * brodawczaka ludzkiego, cervarix *, gardasil *, silgard *	COVID *, koronawirus *, SARS-CoV-2	1430
4.	Vaccination in general without posts related to the COVID-19 pandemic	szczepi *	COVID *, koronawirus *, SARS-CoV-2	70,239

**Table 2:** Mean number of reactions to a posts by type of sentiment to vaccination. Source: original materials.

	Sentiment to Vaccination	Negative	Positive	Neutral
Reactions				
all		426	894	535
like		308	628	268
		(72.3%)	(70.2%)	(50.1%)
love		31	145	23
		(7.3%)	(16.2%)	(4.3%)
sad		15	21	31
		(3.5%)	(2.3%)	(3.8%)
wow		12	6	9
		(2.8%)	(0.7%)	(1.7%)
haha		8	16	116
		(1.9%)	(1.8%)	(21.7%)
angry		52	79	89
		(12.2%)	(8.8%)	(16.6%)
thankful		0	0	0

## Data Availability

The data presented in this study are available within the article.
